# Zonisamide Therapy Reduces Metabolic Consequences and Diminishes Nonalcoholic Fatty Liver Disease in Patients with Epilepsy

**DOI:** 10.3390/jcm10153380

**Published:** 2021-07-30

**Authors:** Chi-Ren Huang, Hung-Yi Chuang, Nai-Ching Chen, Shu-Fang Chen, Chung-Yao Hsu, Yao-Chung Chuang

**Affiliations:** 1 Department of Neurology, Kaohsiung Chang Gung Memorial Hospital, Kaohsiung 83301, Taiwan; suika68@cgmh.org.tw (C.-R.H.); naiging@yahoo.com.tw (N.-C.C.); fangoel@yahoo.com.tw (S.-F.C.); 2 College of Medicine, Chang Gung University, Tao-Yuan 33302, Taiwan; 3 Department of Public Health and Environmental Medicine, College of Medicine, Kaohsiung Medical University Hospital, Kaohsiung Medical University, Kaohsiung 80708, Taiwan; hychuang@gmail.com; 4 Department of Neurology, School of Medicine, College of Medicine, Kaohsiung Medical University Hospital, Kaohsiung Medical University, Kaohsiung 80708, Taiwan; cyhsu61@gmail.com; 5 Department of Biological Science, National Sun Yat-Sen University, Kaohsiung 80424, Taiwan; 6 Institute for Translation Research in Biomedicine, Kaohsiung Chang Gung Memorial Hospital, Kaohsiung 83301, Taiwan

**Keywords:** zonisamide, epilepsy, obesity, metabolic consequences, nonalcoholic fatty liver disease

## Abstract

Antiepileptic drugs that can reduce aberrant metabolism are beneficial for patients. Zonisamide (ZNS) is a chemical with antiepileptic and antioxidant activities. Here, we evaluate the efficacy of ZNS therapy on reducing obesity and decreasing risks of vascular diseases and hepatic steatosis. Clinical and metabolic indicators including body weight, body mass index (BMI), serum lipid profiles, glycated hemoglobin (HbA1c), homocysteine, and an inflammatory marker, high-sensitivity C-reactive protein (hs-CRP), were assessed at baseline and at the end of 12 and 24 weeks of treatment. Nonalcoholic fatty liver disease was evaluated using the hepatic steatosis index (HSI). A body weight reduction of ≥5% was observed in 24.6% and 32.8% of patients after 12 and 24 weeks of ZNS treatment, respectively. After adjusting for age, sex, time, and the corresponding dependent variable at baseline, the generalized estimating equation analysis revealed that the body weight, BMI, serum levels of HbA1c, triglycerides, hs-CRP, and the index for HSI were significantly declined. These results suggest that ZNS provides benefits in patients with obesity and metabolic syndrome at high vascular risk.

## 1. Introduction

Epilepsy is a serious neurological disorder that affects more than 70 million people worldwide [1]. Although most patients with epilepsy have a good prognosis, more than 30% of patients still cannot achieve remission with conventional or newly developed antiepileptic drug (AED) therapies [2,3]. Long-term AED therapy is usually required for patients with refractory epilepsy [2]. As the population ages, the number of elderly patients with epilepsy is increasing. The treatment of elderly patients with epilepsy is complicated due to their medical and neurological comorbidities, drug-drug interactions, increased vascular risks, and the changing pharmaco-kinetics and pharmacodynamics of AEDs [4,5,6,7,8].

Zonisamide (1,2-benzisoxazole-3-methanesulfonamide, ZNS) is a unique chemical with antiepileptic properties [9,10]. It was first approved in Japan for the treatment of epilepsy in 1989 [9,10]. Blockage of voltage-gated sodium and T-type calcium channels was previously suggested as the major mechanisms underlying ZNS activity; however, the precise mechanisms of action remain unclear [9,10]. ZNS is deemed as a broad-spectrum AED with favorable efficacy and is well-tolerated by patients with epilepsy [9,10,11]. It has been approved in many countries as a monotherapy or adjunctive therapy for focal or generalized onset epilepsy in adults and children [9,11,12]. In addition to the antiepileptic property, ZNS is also reported to have antioxidant activities, which may protect neurons from oxidative damage, stabilize the neuronal membranes, and prevent epileptogenic focus formation [13,14]. Recently, ZNS was listed as an initial monotherapy with level A efficacy/effectiveness evidence for adults with partial seizure onset by the International League Against Epilepsy [12].

Pharmacological therapy in patients with epilepsy is usually associated with weight changes that may increase morbidity and drug noncompliance [15]. ZNS and topiramate may lead to body weight loss [12,15,16]; valproic acid, gabapentin, pregabalin, vigabatrin, and carbamazepine may increase weight gain [15,17]. Obesity and metabolic syndromes are associated with increased risks of cardiovascular diseases, cerebrovascular disease, type 2 diabetes, arthritis, cancers, and decreased life expectancy [18], and they are common comorbidities in adult, adolescent, and as pediatric patients with epilepsy [15,19,20,21]. Recently, our team [8,22] and other researchers [6,23,24] have found that long-term AED therapy may increase body weight, body mass index (BMI), metabolic consequences, and oxidative stress in patients with epilepsy, which may further lead to potential vascular risks and acceleration of atherosclerosis [5,8,22,25], particularly in patients who received liver enzyme-inducing AEDs such as phenobarbital, phenytoin, and carbamazepine [5,22,25]. Thus, AEDs that promote weight gain and metabolic consequences should be avoided in patients with epilepsy who are also obese or with metabolic syndromes. Instead, AEDs that are weight-neutral or promote weight loss is suggested to reduce the risk of obesity and complications [5,15].

Body weight reduction is a well-recognized effect of ZNS therapy in patients with epilepsy [11,26,27] or obese adults [28,29,30]. Although enzyme-inducing AEDs may enhance clearance of ZNS, ZNS is not considered as an inducer of the liver enzyme system [10,31]. Since ZNS is a non-enzyme-inducing AED that may reduce body weight [9,10,31], in the present study, we aim to evaluate whether biochemical markers of vascular risk, along with body weight, are reduced with ZNS treatment in patients with epilepsy. Additionally, we want to evaluate whether ZNS therapy attenuates nonalcoholic fatty liver disease by measuring the fatty liver index and hepatic steatosis index (HSI) [32].

## 2. Materials and Methods

### 2.1. Patients and Study Design

This is a prospective case-control study conducted at Kaohsiung Chang Gung Memorial Hospital, a tertiary medical center in Taiwan. The Institutional Ethics Committee of Chang Gung Memorial Hospital approved the study protocol (approval number: 201600466B0), and informed written consent was obtained from all subjects.

From June 2016 to May 2017, 126 adult patients with focal onset epilepsy (aged between 18 and 65 years) who visited to the Epilepsy Outpatient Clinic of Kaohsiung Chang Gung Memorial Hospital were recruited in the present study. Among them, 81 patients (39 females and 42 males) who received ZNS monotherapy or adjunctive therapy were classified into the ZNS-treated group. Forty-five patients (20 females and 25 males) who were not receiving ZNS therapy were recruited as controls. The AEDs used upon recruitment remained unchanged during the study period in both groups.

Subjects having diabetes mellitus (fasting blood sugar ≥126 mg/dL), hypertension, smoking, alcohol use, hepatic diseases, renal insufficiency, pregnancy, major psychiatric disorders, nephrolithiasis history, endocrine diseases, hematological diseases, and autoimmune diseases were excluded. Subjects who took antipsychotic drugs, oral antidiabetic agents, or other medications that could affect blood sugar levels, lipid metabolism, or body weight, or who had deliberately attempted to control their body weight were also excluded. The clinical records of all subjects were reviewed and registered, including age at seizure onset, seizure semiology, seizure etiology, average seizure frequency per month during the previous year, and concomitant AED use. Brain magnetic resonance imaging was performed to exclude confounding progressive brain diseases, such as cerebrovascular diseases or brain tumors.

All patients underwent interviews and physical examinations. Blood samples were collected at baseline (pre-ZNS treatment) for analysis of biochemical markers. Body weight and height were recorded, and BMI was calculated. In the ZNS-treated group, the initial dose of ZNS was 100 mg/day, and it was increased by 100 mg/day every 2 weeks. The target and maintenance doses were dependent on the patient’s tolerability and the drug’s efficacy. During the study period, the dosage and type of AEDs were maintained in both the ZNS-treated and control groups. Twelve and 24 weeks after initiating ZNS treatment, the body weight, height, and BMI were recorded, and the second and third blood samples were collected for analysis of biochemical markers in both groups.

### 2.2. Analysis of Biochemical Markers

Blood samples were collected after overnight fasting and analyzed at the Central Laboratory of our hospital for serum levels of fasting sugar, glycated hemoglobin (HbA1c), high-sensitivity C-reactive protein (hs-CRP), creatinine, aspartate trans-aminase (AST), alanine transaminase (ALT), total homocysteine (tHcy), triglycerides, total cholesterol, high-density lipoprotein cholesterol (HDL-C), and low-density lipoprotein cholesterol (LDL-C).

### 2.3. Measurement of Hepatic Steatosis Index for Nonalcoholic Fatty Liver Disease

A fatty liver index, HSI, was introduced to evaluate nonalcoholic fatty liver disease [32]. HSI was calculated as follows:

HSI = 8 × (ALT/AST ratio) + BMI (+2, if female; +2, if diabetes mellitus)(1)

### 2.4. Statistical Analysis

First, we compared the baseline data between the ZNS-treated and control group patients. Sex was analyzed with the Chi-square test. Continuous variables including age, body weight, BMI, HSI, and serum levels of HbA1c, ALT, AST, creatinine, tHcy, triglycerides, total cholesterol, HDL-C, and LDL-C were compared by Student’s *t*-tests. Paired sample *t*-tests were utilized to compare the baseline biochemical and HSI data after 12 weeks and 24 weeks of ZNS treatment.

Dependent variables, including body weight, BMI, serum levels of HbA1c, triglycerides, total cholesterol, LDL-C, and HSI were measured at baseline and every 12 weeks. We used the generalized estimating equation (GEE) approach to assess the effects of ZNS treatment on body weight, BMI, circulating biochemical markers, and HSI with adjustment for age, sex, time, and the dependent variables at baseline [33]. We also determined whether the dose-dependent effects of ZNS on the dependent variables was estimated by GEE. Statistical analyses were performed using SAS version 9.4, GENMODE procedure (SAS Statistical Institute, Cary, NC, USA). For all analyses, *p* values < 0.05 were considered statistically significant.

## 3. Results

### 3.1. Clinical Characteristics of Patients

**Figure 1:**
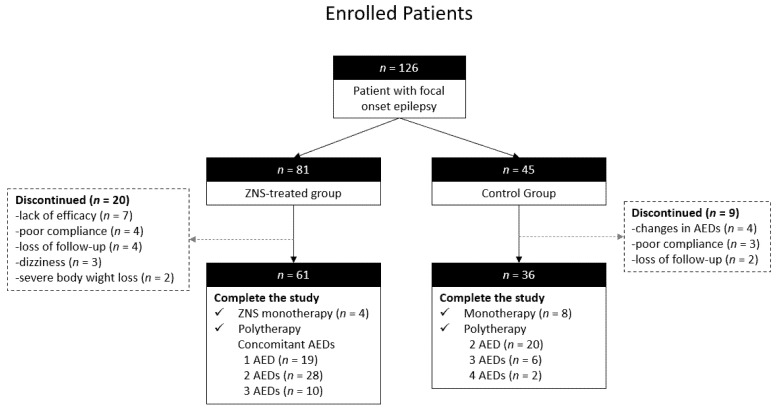
The algorithm of the enrolled patient in the present study. ZNS, zonisamide; AED, antiepileptic drug.

Figure 1 illustrates the algorithm of the selection patient in the present study. Of the 81 patients in the ZNS-treated group, 20 patients (24.7%) discontinued the study. In the control group, nine patients (20.0%) discontinued the study.

Among the 61 ZNS-treated patients, four had newly diagnosed epilepsy, received ZNS monotherapy, and remained seizure-free during the study period. In the others, ZNS was combined with concomitant AEDs included valproic acid (*n* = 28), levetiracetam (*n* = 25), lamotrigine (*n*= 20), carbamazepine (*n* = 16), oxcarbazepine (*n*= 6), phenytoin (*n* = 5), vigabatrin (*n* = 2), phenobarbital (*n* = 2), and pregabalin (*n* = 1). In the control group, concomitant AEDs included levetiracetam (*n* = 19), valproic acid (*n* = 15), carbamazepine (*n* = 13), lamotrigine (*n* = 10), phenytoin (*n* = 7), vigabatrin (*n* = 4), oxcarbazepine (*n* = 3), phenobarbital (*n* = 2), and pregabalin (*n* = 1).

In the ZNS-treated group, the maintenance doses of ZNS were 100 mg/day (*n* = 13), 200 mg/day (*n* = 17), 300 mg/day (*n* = 14), 400 mg/day (*n* = 14), and 500 mg/day (*n* = 3). Among them, 18.0% (11/61) of patients were seizure-free and 45.9% (28/61) achieved a ≥50% responder rate within 24 weeks of the study period.

The demographic data and results of the circulating biochemical marker analyses are listed in Table 1. Age, sex, body weight, BMI, and most biochemical markers were not significantly different between the ZNS-treated and control groups at baseline. Triglyceride, total cholesterol, and LDL-C levels were significantly higher in the ZNS-treated group as compared with controls. These factors may have been biased due to patient selection.

### 3.2. Changes in Body Weight, BMI, Circulating Biochemical Markers, and HSI after ZNS Therapy

Table 2 lists the change in body weight, BMI, circulating biochemical markers, and HSI after 12 and 24 weeks of ZNS treatment. The average body weight decreased 2.48% and 2.75% after 12 and 24 weeks of ZNS treatment, respectively. A body weight reduction was observed in 55.7% (34/61) of patients after 12 weeks of ZNS treatment and 65.6% (40/61) of patients after 24 weeks of ZNS treatment. Weight loss of at least 5% was observed in 24.6% (15/61) and 32.8% (20/61) of patients after 12 and 24 weeks of ZNS treatment, respectively. Regarding BMI, there was 2.56% and 2.82% reduction after 12 and 24 weeks of ZNS treatment, respectively. The percentage of patients with a reduction in BMI of ≥5% after 12 and 24 weeks of ZNS treatment was similar to that of body weight loss (24.6% and 32.8%, respectively).

The statistical analysis (Table 2) revealed that the serum levels of hs-CRP, HbA1c, triglycerides, and LDL-C were significantly decreased after 12 weeks of ZNS treatment compared with baseline values. At the end of week 24, the serum levels of hs-CRP, HbA1c, and triglycerides remained significantly decreased. 

To determine whether body weight loss was associated with reduced nonalcoholic fatty liver disease, we used a fatty liver index, HSI [34], to evaluate nonalcoholic fatty liver disease. Interestingly, the serum levels of AST, ALT, and HSI were significantly reduced as compared with the baseline values.

### 3.3. GEE Analysis of Body Weight, BMI, Circulating Biochemical Markers, and HSI after ZNS Therapy

We performed GEE to estimate whether body weight, BMI, HSI, HbA1c, triglycerides, total cholesterol, LDL-C, and hs-CRP were dependent on ZNS treatment after adjusting for age, sex, time, and the dependent variables at baseline (Table 3). We found that body weight, BMI, HSI, and serum levels of HbA1c, triglycerides, and hs-CRP showed a significant decline after ZNS treatment. In contrast, the total cholesterol and LDL-C levels showed no significant difference (*p* > 0.05). After adjustments, the GEE approach revealed that the decreasing trends in body weight, BMI, HSI, HbA1c, triglycerides, and hs-CRP were not dependent on ZNS doses from 100 to 500 mg (data not shown).

### 3.4. Effect Plots of ZNS Therapy by GEE Method with Adjustment for Sex, Age, and De-Pendent Variables at Baseline

**Figure 2:**
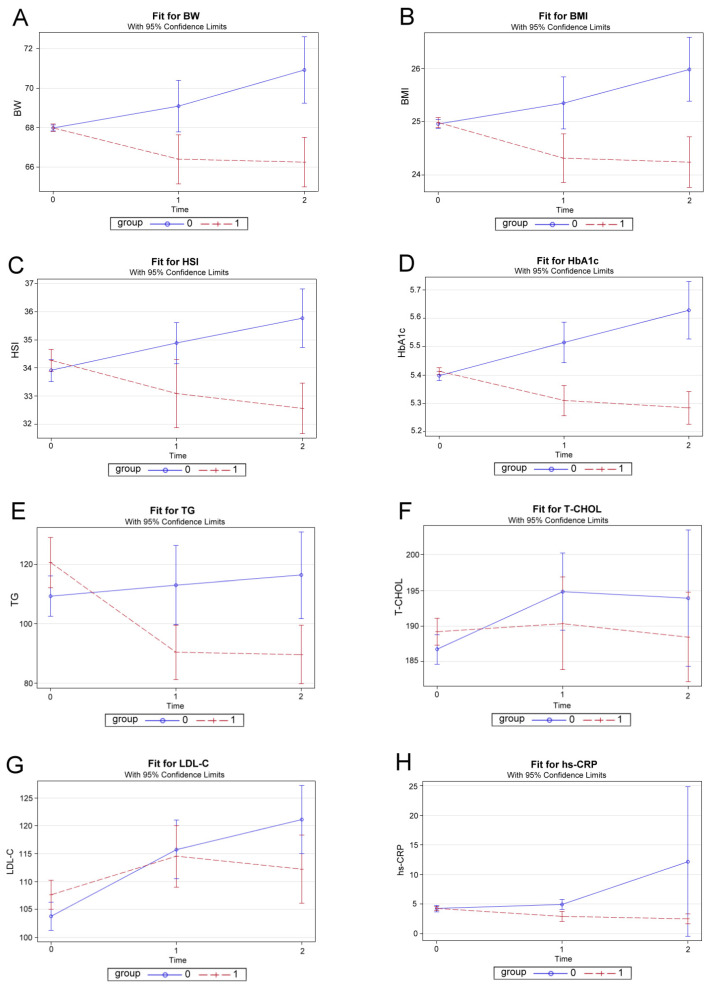
Plots of effects of zonisamide therapy, time fitted by generalized estimating equation (GEE) and adjusted for sex, age, and dependent variables (Y-axes) at baseline. (**A**) body weight; (**B**) body mass index; (**C**) hepatic steatosis index; (**D**) glycosylated hemoglobin; (**E**) triglycerides; (**F**) total cholesterol; (**G**) low-density lipoprotein cholesterol; (**H**) high-sensitivity C-reactive protein. Group 0, Control group; Group 1, Zonisamide group; time 0, baseline; time 1, 12 weeks; time 2, 24 weeks. We used GEE analysis rather than the two-way ANOVA because it was more appropriate for our data. BW, body weight; BMI, body mass index; HSI, hepatic steatosis index; HbA1c, glycosylated hemoglobin; TG, triglycerides; T-CHOL, total cholesterol; LDL-C, low-density lipoprotein cholesterol; hs-CRP, high-sensitivity C-reactive protein.

The effects of ZNS therapy at baseline (0), 12 weeks after treatment (1), and 24 weeks after treatment (2) is illustrated in Figure 2. The body weight, BMI, hs-CRP, and HbA1c were significantly decreased among the treated patients in the first 12 weeks, while these variables showed an increasing trend throughout the therapy duration in the control group (Figure 2A,B,D,H). The HSI and triglycerides were significantly decreased in the ZNS-treated group as compared to the control group after 24 weeks (Figure 2C,E). The effects on total cholesterol and LDL-C showed decreasing trends in the ZNS-treated group, but statistical significance was not achieved (Figure 2F,G).

## 4. Discussion

The present study evaluated the changes in body weight, BMI, and vascular risk factors in patients with epilepsy who received ZNS therapy. After adjusting for age, sex, time, and the dependent variables at baseline using GEE analysis, our results confirmed that ZNS therapy significantly reduced body weight and BMI. Additionally, ZNS therapy might reduce vascular risk factors, including serum levels of HbA1c, triglycerides, and hs-CRP. Interestingly, we demonstrated that patients with epilepsy had diminished nonalcoholic fatty liver disease with ZNS therapy, as assessed by HSI [32].

Evidence has suggested that patients with epilepsy have a higher risk of obesity and metabolic syndromes than the general population [19,20,21,34,35]. In the central nervous system, epilepsy can potentially affect hypothalamic neuroendocrine control of energy homeostasis that contributes to obesity [20,36]. In the present study, we demonstrated that ZNS therapy produces significant body weight loss alongside decreased BMI in patients with epilepsy. In a retrospective chart analysis study with 103 epilepsy patients [28], the authors suggested that body weight loss was not correlated with daily ZNS dosage and it was reversible after discontinuation of ZNS treatment [27,30]. This was consistent with our results of GEE analysis, where the body weight loss after ZNS therapy was not correlated with daily ZNS dosage. Nevertheless, further studies with a larger sample size are required to verify this correlation.

The precise mechanism of ZNS-induced body weight loss remains unknown. According to Gadde et al., the effect of weight loss might be related to brain serotonin and dopamine systems [28]. ZNS is a sulfonamide that acts as a weak carbonic anhydrase inhibitor in vivo [10], which is comparable to acetazolamide, another medication for treating epilepsy [37]. However, the carbonic anhydrase inhibitory activity of acetazolamide was not suggested as the primary antiepileptic mechanism of ZNS [10]. Carbonic anhydrase inhibitors of the sulfonamide and sulfamate type are clinically emerging drugs for the treatment of obesity, epilepsy, hypertension, glaucoma, and high-altitude disease [16,38]. Evidence has demonstrated that some AEDs, such as ZNS and topiramate, potently inhibit both cytosolic and mitochondrial carbonic anhydrases involved in lipogenesis [38,39,40]. Therefore, we proposed that ZNS-induced weight loss in patients is partly related to inhibition of the mitochondrial carbonic anhydrases involved in lipogenesis [38,40]. Our analysis demonstrated statistically significant decreases in triglycerides after ZNS treatment in patients with epilepsy. On the other hand, the profile of cholesterol, including the total cholesterol, HDL-C, and LDL-C levels showed no significant difference between the ZNS-treated and control groups. We suggest that the improved dyslipidemia with reduced triglyceride levels after ZNS treatment is associated with loss of body weight [41]. Decreased body weight after ZNS treatment may be accompanied by reduced triglyceride levels, but changes in total cholesterol and LDL-C levels showed no significant difference between the two groups. This effect of reduced triglyceride levels may be similar to the effects of a lifestyle intervention in individuals with type 2 diabetes [42]. Perhaps the inhibition of lipogenesis utilizing the mitochondrial carbonic anhydrase inhibitory activity of ZNS partly contributes to the decreased triglyceride levels [38,39]. Further studies are necessary to corroborate these observations.

In the present study, HbA1c levels decreased significantly after 12 and 24 weeks of ZNS treatment. In a randomized controlled trial [29], obese adults who received 400 mg/day of ZNS therapy had statistically lower body weights and HbA1c levels than the control group. Acetazolamide has been used in the treatment of early-stage diabetes [43], which may be related to its diuretic activity in the renal proximal convoluted tubule and highly efficient carbonic anhydrase inhibition. In the present study, we noted that the serum levels of creatinine were significantly increased after 12 and 24 weeks of ZNS treatment. This finding is consistent with that of a previous report [29], where serum creatinine levels were also increased after ZNS treatment of obese adults. The mechanism causing decreased serum HbA1c levels in ZNS-treated patients is unclear. We propose that the decreased HbA1c levels are related to the central hypothalamic effect on appetite [28] and the decrease in triglycerides that is contributed to the effect of calcium inhibition on mitochondrial function and lipogenesis [38,39,40]. Additionally, in patients with glucose transporter 1 deficiency, the benefit effect of ZNS on seizure control has been reported in the literatures [44]. Therefore, the measurements of insulin or c peptide levels may help us understand the effect of ZNS on glucose transporters and insulin resistance. Nevertheless, more studies are required to verify this hypothesis.

Nonalcoholic fatty liver disease is characterized by hepatic lipid accumulation that is not caused by excessive alcohol intake and is the leading cause of chronic liver disease worldwide [45,46]. The present study took advantage of a simple screening tool, HIS [32], to measure nonalcoholic fatty liver disease in patients with epilepsy who received ZNS therapy. HSI is significantly correlated with ultrasound steatosis grade [32]. Our results provide novel evidence demonstrating that decreased HSI is accompanied by decreased serum levels of AST and ALT after ZNS treatment. Therefore, ZNS treatment may diminish nonalcoholic fatty liver disease and improve liver function. Furthermore, non-alcoholic fatty liver disease is considered as a feature of metabolic syndrome and is closely associated with obesity, dyslipidemia, insulin resistance, and diabetes [32,45,46]. In obesity and over-nutrition, hepatic fatty acid metabolism may be altered, leading to the accumulation of triglycerides within hepatocytes that cause nonalcoholic fatty liver disease [45,47]. The pathophysiology of nonalcoholic fatty liver disease and its progression is influenced by multiple factors (environmental and genetics), where oxidative stress was suggested to play a primary role as the starting point of the hepatic and extrahepatic damage [48]. ZNS was reported to have antioxidant activities, including inhibiting lipid peroxidation, decreasing production of nitric oxide, and scavenging hydroxyl radicals [13,49]. The antioxidant properties of ZNS may contribute to the neuroprotective characteristics of this drug [14]. Therefore, the diminished nonalcoholic fatty liver disease after ZNS treatment may be related to reduced body weight and improved dyslipidemia, particularly decreased triglyceride levels, and oxidative stress. Nevertheless, advanced studies for nonalcoholic fatty liver disease, such as ultrasound of the liver, magnetic resonance imaging, or fibroscan, which could provide additional information, may be required in future work.

Patients with epilepsy who have received long-term AED therapy, particularly en-zyme-inducing AEDs, manifested with increased serum levels of hs-CRP, a circulating biomarker of inflammation [5,8,22,24,50]. This implies that these patients with increased hs-CRP have chronic, low-grade inflammation in their vessel walls and an increased atherosclerotic risk [50,51]. Our results showed that the circulating levels of hs-CRP decreased after ZNS treatment. This suggests that the inflammation cascade was decreased after ZNS therapy. Because the serum levels of hs-CRP are related to obesity, diabetes mellitus, and dyslipidemia [50,51], we propose that the decreased hs-CRP is associated with the reduced body weight and ameliorated dyslipidemia in patients with epilepsy with ZNS treatment. 

We are aware of the potential adverse effects of ZNS treatment in patients with epilepsy. In the present study, we did not observe other adverse effects except for dizziness and body weight loss. The most common adverse events of ZNS are fatigue, somnolence, headache, dizziness, nausea, and anxiety [9,11,26]. Although these adverse effects are usually mild and well tolerated, serious adverse effects, including renal stones and metabolic acidosis, should be a concern in high-risk patients.

There are some limitations in the present study. First, this is a case-control study. A less conclusive statement may be made with a case-control study as compared with a double-blind design. Nevertheless, in our cases, ethical issues may arise if we administered a placebo to patients with epilepsy instead of AEDs. Second, waist circumference is well correlated with visceral fat and is a useful parameter for metabolic disorders. However, as the waist circumference examination is not routinely performed in our department, the data of waist circumference was incomplete. Third, in the present study, the number of patients receiving ZNS monotherapy was relatively small. We could not exclude the possibility of interaction effect of ZNS with other AEDs on the reduction of body weight or other parameters. Overall, further studies are warranted to address these issues.

## 5. Conclusions

In conclusion, ZNS therapy in patients with epilepsy may effectively reduce body weight and decrease metabolic consequences, including serum levels of HbA1c, triglycerides, and hs-CRP. ZNS has the ability of carbonic anhydrase inhibition, and can be a potential therapeutic target for obesity. Additionally, our results provide novel evidence for diminished nonalcoholic fatty liver disease after ZNS treatment. This information indicates that ZNS may provide benefits in patients with epilepsy who are obese and have metabolic syndromes, particularly in elderly patients at elevated risk of vascular diseases.

## Figures and Tables

**Table 1:** Comparison of ZNS-treated and control patients at baseline.

	ZNS Group	Control Group	*p*
Age (years)	40.26 ± 10.90	40.47 ± 9.64	0.924
Sex (female; male)	27; 34	16; 20	0.999 ^a^
Body weight (kg)	67.92 ± 14.76	68.10 ± 13.92	0.952
BMI (kg/m^2^)	25.185 ± 4.468	24.620 ± 4.413	0.538
hs-CRP (mg/L)	4.551 ± 10.089	3.818 ± 5.734	0.691
HbA1c (%)	5.45 ± 0.38	5.33 ± 0.32	0.109
Creatinine (mg/dL)	0.796 ± 0.244	0.738 ± 0.159	0.163
AST (U/L)	20.97 ± 13.24	19.19 ± 6.43	0.454
ALT (U/L)	23.10 ± 15.52	19.944 ± 16.372	0.346
HSI	34.806 ± 6.969	32.996 ± 5.571	0.188
tHcy (μmol/L)	16.561 ± 19.443	15.813 ± 12.782	0.837
Triglycerides (mg/dL)	127.95 ± 94.48	97.08 ± 57.23	0.048
Cholesterol (mg/dL)			
Total	194.02 ± 36.50	178.61 ± 27.50	0.031
HDL-C	58.48 ± 17.46	64.08 ± 17.89	0.133
LDL-C	112.18 ± 38.11	96.11 ± 26.74	0.028

Except for sex, values are expressed as mean ± standard deviation. ZNS, zonisamide; BMI, body mass index; hs-CRP, high-sensitivity C-reactive protein; HbA1c, glycated hemoglobin; AST, aspartate transaminase; ALT, alanine transaminase; HSI, hepatic steatosis index; tHcy, total homocysteine; HDL-C, high-density lipoprotein cholesterol; LDL-C, low-density lipoprotein cholesterol. ^a^ Chi-square test, all others: Student’s *t*-test.

**Table 2:** Change in factors from baseline in zonisamide-treated patients.

	12 Weeks		24 Weeks	
	Difference Mean ± SD	*p*	Difference Mean ± SD	*p*
Body weight (kg)	−1.61 ± 5.10	0.017 *	−1.75 ± 5.31	0.013 *
BMI (kg/m^2^)	−0.670 ± 1.909	0.008 *	−0.747 ± 2.031	0.006 *
hs-CRP (mg/L)	−1.340 ± 3.567	0.005 *	−1.753 ± 3.528	<0.001 *
HbA1c (%)	−0.10 ± 0.22	0.001 *	−0.13 ± 0.24	<0.001 *
Creatinine (mg/dL)	0.036 ± 0.114	0.017 *	0.048 ± 0.115	0.002 *
AST (U/L)	−2.31 ± 11.59	0.125	−3.25 ± 12.11	0.041 *
ALT (U/L)	−3.46 ± 14.21	0.062	−5.33 ± 13.36	0.003 *
HSI	−1.173 ± 5.646	0.110	−1.701 ± 4.448	0.004 *
tHcy (μmol/L)	−2.065 ± 17.838	0.369	−2.664 ± 18.669	0.269
Triglycerides (mg/dL)	−30.34 ± 62.02	<0.001 *	−31.07 ± 65.98	0.001 *
Cholesterol (mg/dL)				
Total	1.15 ± 27.79	0.748	−0.75 ± 26.89	0.827
HDL-C	−1.80 ± 12.61	0.268	−1.57 ± 12.61	0.333
LDL-C	6.89 ± 26.58	0.048 *	4.57 ± 28.99	0.223

SD, standard deviation; BMI, body mass index; hs-CRP, high-sensitivity C-reactive protein; HbA1c, glycated hemoglobin; AST, aspartate transaminase; ALT, alanine transaminase; HSI, hepatic steatosis index; tHcy, total homocysteine; HDL-C, high-density lipoprotein cholesterol; LDL-C, low-density lipoprotein cholesterol. * *p* < 0.05, paired sample *t*-test.

**Table 3:** Generalized estimating equation for dependent variables in the zonisamide-treated and control groups ^a^.

	**Body Weight (kg)**			**BMI (kg/m^2^)**			**Hepatic Steatosis Index (HSI)**			**HbA1c (%)**		
**Effect**	**Estimate**	**SE**	***p***	**Estimate**	**SE**	***p***	**Estimate**	**SE**	***p***	**Estimate**	**SE**	***p***
Intercept	0.220	1.648	0.894	0.746	0.774	0.336	7.522	1.377	0.009	0.814	0.170	<0.0001
ZNS group	−2.459	0.502	<0.001	−0.914	0.228	<0.001	−1.517	0.414	<0.001	−0.177	0.032	<0.0001
Control group												
12 weeks	−0.603	0.594	0.311	−0.276	0.185	0.137	−0.380	0.485	0.434	−0.022	0.025	0.380
24 weeks	−0.006	0.594	0.992	−0.089	0.216	0.679	−0.383	0.485	0.430	0.004	0.032	0.899
Time = 0 week												
Sex (male vs. female)	1.183	0.574	0.042	0.436	0.247	0.077	0.864	0.405	0.033	0.040	0.026	0.124
Age (years)	0.047	0.026	0.050	0.020	0.010	0.040	0.003	0.192	0.859	0.000	0.001	0.738
Baseline ^b^	0.982	0.020	<0.001	0.951	0.029	<0.001	0.790	0.313	<0.001	0.863	0.034	<0.001
	**Triglyceride (mg/dL)**			**Total Cholesterol (mg/dL)**			**LDL-C (mg/dL)**			**hs-CRP (mg/L)**		
**Effect**	**Estimate**	**SE**	***p***	**Estimate**	**SE**	***p***	**Estimate**	**SE**	***p***	**Estimate**	**SE**	***p***
Intercept	45.425	7.869	<0.001	16.072	12.164	0.186	11.016	5.735	0.055	4.838	3.802	0.203
ZNS group	−12.264	4.819	0.011	−2.198	3.169	0.488	−1.752	2.281	0.442	−3.869	1.717	0.024
Control group												
12 weeks	−17.701	5.966	0.003	3.732	2.494	0.135	8.773	2.631	0.001	−0.583	2.030	0.774
24 weeks	−16.907	6.447	0.009	2.196	2.888	0.447	9.299	2.631	0.000	1.847	2.030	0.363
Time = 0 week												
Sex (male vs. female)	17.142	4.775	0.000	−1.393	2.960	0.638	1.218	2.163	0.574	2.168	1.682	0.198
Age (years)	−0.063	0.163	0.701	0.465	0.174	0.008	0.410	0.104	<0.001	−0.080	0.081	0.321
Baseline ^b^	0.616	0.035	<0.001	0.827	0.054	<0.001	0.745	0.032	<0.001	0.910	0.097	<0.001

SE, standard error; ZNS, zonisamide; BMI, body mass index; HbA1c, glycated hemoglobin; LDL-C, low-density lipoprotein cholesterol; hs-CRP, high-sensitivity C-reactive protein. ^a^ The effect between groups was estimated after adjusting for sex, age, time, and the dependent variable at baseline. ^b^ Baseline: dependent variable at baseline.

## Data Availability

The data used to support the findings of this study are included within the article.
